# Recovery and stable persistence of chloroquine sensitivity in *Plasmodium falciparum* parasites after its discontinued use in Northern Uganda

**DOI:** 10.1186/s12936-020-03157-0

**Published:** 2020-02-18

**Authors:** Betty Balikagala, Miki Sakurai-Yatsushiro, Shin-Ichiro Tachibana, Mie Ikeda, Masato Yamauchi, Osbert T. Katuro, Edward H. Ntege, Makoto Sekihara, Naoyuki Fukuda, Nobuyuki Takahashi, Shouki Yatsushiro, Toshiyuki Mori, Makoto Hirai, Walter Opio, Paul S. Obwoya, Denis A. Anywar, Mary A. Auma, Nirianne M. Q. Palacpac, Takafumi Tsuboi, Emmanuel I. Odongo-Aginya, Eisaku Kimura, Martin Ogwang, Toshihiro Horii, Toshihiro Mita

**Affiliations:** 1 Department of Tropical Medicine and Parasitology, School of Medicine, Juntendo University, 2-1-1 Hongo, Bunkyo-ku, Tokyo 113-0033 Japan; 2 Department of International Affairs and Tropical Medicine, School of Medicine, Tokyo Women’s Medical University, 8-1 Kawada-cho, Shinjuku-ku, Tokyo 162-8666 Japan; 3 Mildmay Uganda, Nazibwa Hill, Lweza, P.O. Box 24985, Kampala, Uganda; 4 Division of Malaria Research, Proteo-Science Center, Ehime University, 3 Bunkyo-cho, Matsuyama, Ehime 790-8577 Japan; 5 Health Research Institute, National Institute of Advanced Industrial Science and Technology (AIST), 2217-14 Hayashi-cho, Takamatsu, Kagawa 761-0395 Japan; 6 St. Mary’s Hospital Lacor, P.O. Box 180, Gulu, Uganda; 7 Faculty of Science, Gulu University, P.O. Box 166, Gulu, Uganda; 8 Department of Malaria Vaccine Development, Research Institute for Microbial Diseases, Osaka University, 3 Yamadaoka, Suita, Osaka 565-0871 Japan; 9 Institute of Tropical Medicine, Nagasaki University, 1-12-4 Sakamoto, Nagasaki, Nagasaki 852-8523 Japan

**Keywords:** Chloroquine sensitivity, Persistent recovery, *Plasmodium falciparum*, Uganda

## Abstract

**Background:**

Usage of chloroquine was discontinued from the treatment of *Plasmodium falciparum* infection in almost all endemic regions because of global spread of resistant parasites. Since the first report in Malawi, numerous epidemiological studies have demonstrated that the discontinuance led to re-emergence of chloroquine-susceptible *P. falciparum*, suggesting a possible role in future malaria control. However, most studies were cross-sectional, with few studies looking at the persistence of chloroquine recovery in long term. This study fills the gap by providing, for a period of at least 6 years, proof of persistent re-emergence/stable recovery of susceptible parasite populations using both molecular and phenotypic methods.

**Methods:**

Ex vivo drug-susceptibility assays to chloroquine (n = 319) and lumefantrine (n = 335) were performed from 2013 to 2018 in Gulu, Northern Uganda, where chloroquine had been removed from the official malaria treatment regimen since 2006. Genotyping of *pfcrt* and *pfmdr1* was also performed.

**Results:**

Chloroquine resistance (≥ 100 nM) was observed in only 3 (1.3%) samples. Average IC_50_ values for chloroquine were persistently low throughout the study period (17.4–24.9 nM). Parasites harbouring *pfcrt* K76 alleles showed significantly lower IC_50_s to chloroquine than the parasites harbouring K76T alleles (21.4 nM vs. 43.1 nM, p-value = 3.9 × 10^−8^). Prevalence of K76 alleles gradually increased from 71% in 2013 to 100% in 2018.

**Conclusion:**

This study found evidence of stable persistence of chloroquine susceptibility with the fixation of *pfcrt* K76 in Northern Uganda after discontinuation of chloroquine in the region. Accumulation of similar evidence in other endemic areas in Uganda could open channels for possible future re-use of chloroquine as an option for malaria treatment or prevention.

## Background

Since the late 1940s, chloroquine was the mainstay for the treatment of *Plasmodium falciparum* infection. Heavy use of chloroquine, however, led to the emergence of *P. falciparum* parasites resistant to chloroquine in Southeast Asia and South America. The resistant parasites that first emerged in Southeast Asia spread to East Africa (Tanzania and Kenya) by 1980 [1, 2] and eventually across the malaria endemic regions of Africa [3]. Chloroquine was, therefore, withdrawn/discontinued for routine treatment of *P. falciparum* malaria in nearly all malaria endemic regions. However, with widespread discontinued use, numerous molecular-epidemiological studies showed that there was return of chloroquine susceptibility in *P. falciparum* field isolates [4]. This is supported by ex vivo [5–16] and in vivo drug-susceptibility studies [5, 17, 18]. Findings suggest that chloroquine might be re-used in the future as an option for the treatment and/or chemoprophylaxis on the condition that chloroquine sensitivity is maintained in the area. As the parasite is “skilled” in evading anti-malarial treatments, continuous surveillance on longitudinal persistence of chloroquine susceptibility by molecular and phenotypic analysis [8, 16, 18, 19] is needed.

In Uganda, first-line treatment for uncomplicated malaria was changed from chloroquine to chloroquine plus sulfadoxine/pyrimethamine in 2000, then again changed to artemether–lumefantrine in 2006 [20]. Several studies in different regions in Uganda reported high prevalence of ex vivo chloroquine resistant parasites (IC_50_s ≥ 100 nM) accompanied with high prevalence of lysine to threonine change at position 76 (K76T) in *pfcrt* [21–24]. However, a recent study showed recovery of chloroquine susceptibility in Eastern Uganda, Tororo; average IC_50_s decreased from 248 nM in 2010–2013 to 33 nM in the community and 57 nM in the hospital setting in 2016 [10]. Here, to investigate whether chloroquine sensitivity also recovered in other regions in Uganda and, if so, to examine the persistence of chloroquine sensitivity, ex vivo drug susceptibility studies for a 6-year period since 2013 in Gulu, Northern Uganda were performed. Results show that chloroquine susceptibility stably persisted during the study period with a significant decrease and eventual absence of the chloroquine resistant K76T alleles in *pfcrt*.

## Methods

### Study site

**Fig. 1:**
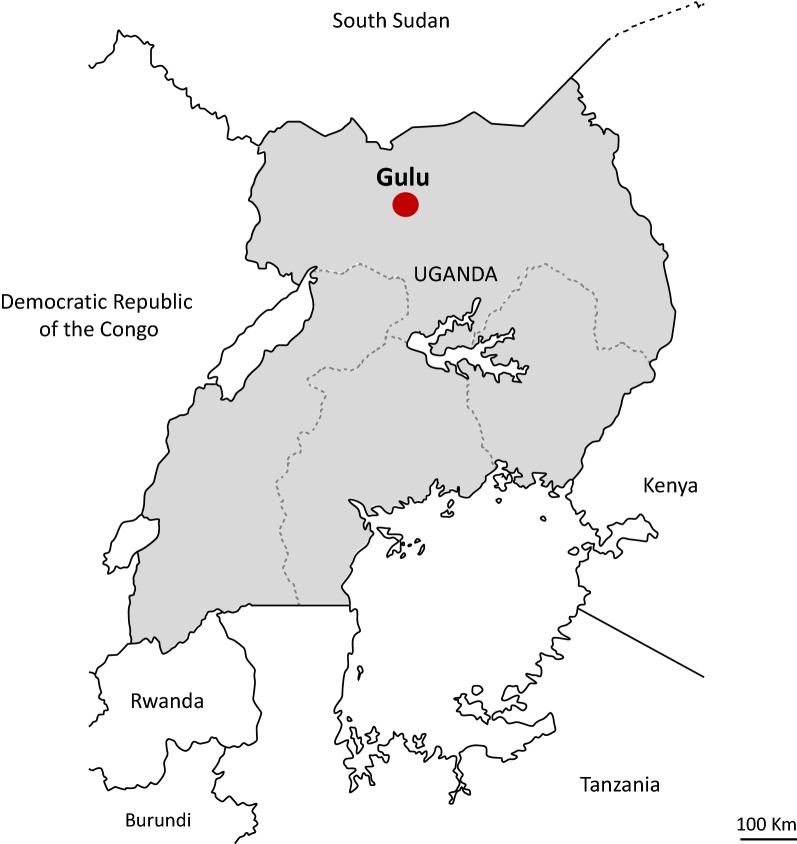
Study site, Gulu (red circle), Northern Uganda

A comprehensive drug susceptibility assessment was conducted at St. Mary’s Hospital Lacor in Gulu, Northern Uganda (Fig. 1) from 2013 to 2018 [25, 26]: Oct–Nov 2013, May–Jun and Oct–Nov 2014, May–Jun and Oct 2015, Jun–Jul and Oct–Nov 2016, Jun 2017 and Jun 2018. Average temperature in the studied area is 24.6 °C and average annual rainfall is about 1507 mm with two rainy seasons; a smaller peak in April–May (average rainfall 150 mm) and a heavier peak in August–September (average rainfall 234 mm) [27]. *Plasmodium falciparum* is the most prevalent species and is mainly transmitted by *Anopheles funestus* and *Anopheles gambiae* as the major vectors.

Malaria control programmes in the studied region include vector control by long-lasting insecticidal nets (LLINs) and indoor residual spraying (IRS), artemisinin-based combination therapy (ACT) together with improved diagnosis, management of severe malaria, and intermittent preventive treatment of malaria during pregnancy. These control measures were performed with funding from the Global Fund, USAID/PMI, DFID, World Vision and other partners [28]. Mass distribution of LLINs was first implemented in 2009–2010, which continued until 2013–2014. Through these extensive efforts, malaria burden was effectively reduced from 72% in 2009 to 29% in 2014 [27].

### Patients

Initial screening was performed using RDT (SD BIOLINE Malaria Ag P.f/Pan test, Abbott, USA) for 1575 symptomatic patients who visited St. Mary’s Hospital Lacor. Inclusion criteria were: (a) patients that are *P. falciparum* positive by RDT and microscopy, (b) aged ≥ 6 months, and (c) with no history of taking anti-malarial drug(s) within 2 weeks before enrollment. Patients fulfilling the inclusion criteria were enrolled after obtaining written informed consent from the patients or parent/guardian(s). For children aged 7 to 17 years, separate assent was also obtained.

Ethical approval for this study was obtained from the Lacor Hospital Institutional Research and Ethics Committee (Ref; LHIREC 021/09/13), the Uganda National Council for Science and Technology (Ref; HS 1395), and Juntendo Research and Ethics Committee (Ref; 14-169).

### Sample collection and ex vivo susceptibility assay for chloroquine and lumefantrine

Blood samples of approximately 100–500 µL (< 2 years old), and 1 mL (≥ 2 years old) were collected by peripheral venipuncture or finger prick and immediately transferred to the laboratory adjacent to the hospital. Thick and thin blood smears stained for 30 min with 2% Giemsa solution were used to determine parasitaemia.

At every visit from 2013 to 2018 (a total of nine times sampling period), ex vivo drug susceptibility studies were performed. Ex vivo susceptibility was evaluated for chloroquine and lumefantrine for the samples with parasitemia ≥ 0.05% as previously reported [29]. Parasite culture was incubated in the presence of chloroquine (25–1600 nM) or lumefantrine (1.25–80 nM) at 37 °C for 72 h in a gas atmosphere of 5% CO_2_, 5% O_2_ (AnaeroPack malaria culture system, Mitsubishi Gas Chemical Co. Inc., Tokyo, Japan). Laboratory-maintained 3D7 clone was used for quality evaluation of pre-dosed drug plates. Parasite culture without antimalarials served as control. To evaluate parasite growth, thick smears were made from drug free culture after 72 h of incubation and the number of schizonts counted. If less than 5 schizonts were seen per field, the test samples included in that plate were not used for further analysis. Drug sensitivity was assessed using an enzyme-linked immunosorbent assay (ELISA) that quantifies parasite histidine-rich protein-2 (HRP-2) [30]. The effective concentration needed to inhibit *P. falciparum* growth by 50% (IC_50_) was determined by non-linear regression using an online ICEstimator software (http://www.antimalarial-icestimator.net) [31]. The quality of the ex vivo drug assay was evaluated based upon the level of fitness to the expected shape of the curve obtained by the inhibitory sigmoid Emax model [31].

### *pfcrt* and *pfmdr1* genotyping

Polymorphisms at amino acid positions 72–76 in the *P. falciparum* chloroquine resistance transporter gene (*pfcrt)* were determined by direct sequencing. In the *P. falciparum* multidrug resistance-1 (*pfmdr1*) gene, polymorphisms at codons 86, 184, 1034, 1042 and 1246 were determined by direct sequencing and/or restriction fragment length polymorphism (RFLP) analysis, as previously described [29, 32]. For direct sequencing, initial and nested PCR was done with PrimeSTAR Max DNA Polymerase (Takara Bio Inc., Japan) in 10 μL reaction mixture containing 1 μL of DNA template and 0.5 μM of each primer set. Excess primers and unincorporated nucleotides from the nested PCR product were enzymatically removed with ExoSAP-IT Kit (Amersham Biosciences, Buckinghamshire, UK) and direct sequence was performed (96 °C for 1 min, 25 cycles of 96 °C for 30 s, 50 °C for 30 s and 60 °C for 4 min, and a final cycle at 60 °C for 1 min) with a BigDye Terminator v1.1 cycle sequencing kit in the Applied Biosystems 3130/3130xL genetic analyzer (Life Technologies, Carlsbad, California, USA). Samples with overlapping peaks of at least 50% in height were considered harboring mixed genotypes.

### Full sequencing of *pfcrt*

The full sequence of *pfcrt* was obtained either by whole-genome sequencing (n = 17) or target sequencing (n = 39). Whole genome sequence data was previously reported [26]. In brief, Acrodisc filters (Pall Corporation, New York, NY, USA) was used to reduce the extent of human DNA contamination from blood samples. Approximately 1–1.5 Gb of data per sample were obtained using Illumina instruments (Miseq and Hiseq 2000). Single-nucleotide polymorphisms were called at all genomic positions with > 80% frequency of > 10 reads support.

For target sequencing, the DNA fragment of a genomic region coding for *pfcrt* gene was amplified by PCR with primers (*Pfcrt*-F: 5′-TAC TTT CCC AAG TTG TAC TGC TTC TAA GCT-3′, *Pfcrt*-R: 5′-TTT ACC TAT TTA TCA AAA CAC CAA AAG GGA-3′), which covers the whole DNA sequence of *pfcrt* gene. PCR was performed with PrimeSTAR GXL DNA Polymerase (Takara Bio Inc., Japan) in 5 μL reaction mixture containing 1 μL of DNA solution and 0.25 μM of primer set. PCR conditions consisted of denaturation at 98 °C for 10 s, followed by 40 cycles of amplification (98 °C for 10 s, 60 °C for 15 s, and 68 °C for 5 min), with a final elongation period of 68 °C for 5 min. PCR products were diluted with 5 μL of pure water, electrophoresed in 2% agarose gel and stained with ethidium bromide. The PCR products were then purified with the ExoSAP-IT reagent (Affymetrix, USA). Libraries were prepared from the purified PCR products with Nextera XT DNA Library Prep Kit (Illumina, USA). The libraries were sequenced by MiSeq (Illumina) with the paired-end method and 250 bp of read length. The reads were also used to map the *pfcrt* gene sequence of *P. falciparum* 3D7 as a reference and assembled a single contiguous sequence by CLC Genomics Workbench (Qiagen). All sequences were deposited in the DNA Data Bank of Japan (DDBJ) with accession numbers LC498195–LC498250.

### Statistical analysis

All statistical analyses were performed using R software (version 3.6.1). Data was analyzed using Kruskall Wallis test, Wilcoxon rank sum test, and Jonckheere-Terpstra test. p values < 0.05 were considered statistically significant.

## Results

### Ex vivo drug susceptibility of chloroquine and lumefantrine

**Fig. 2:**
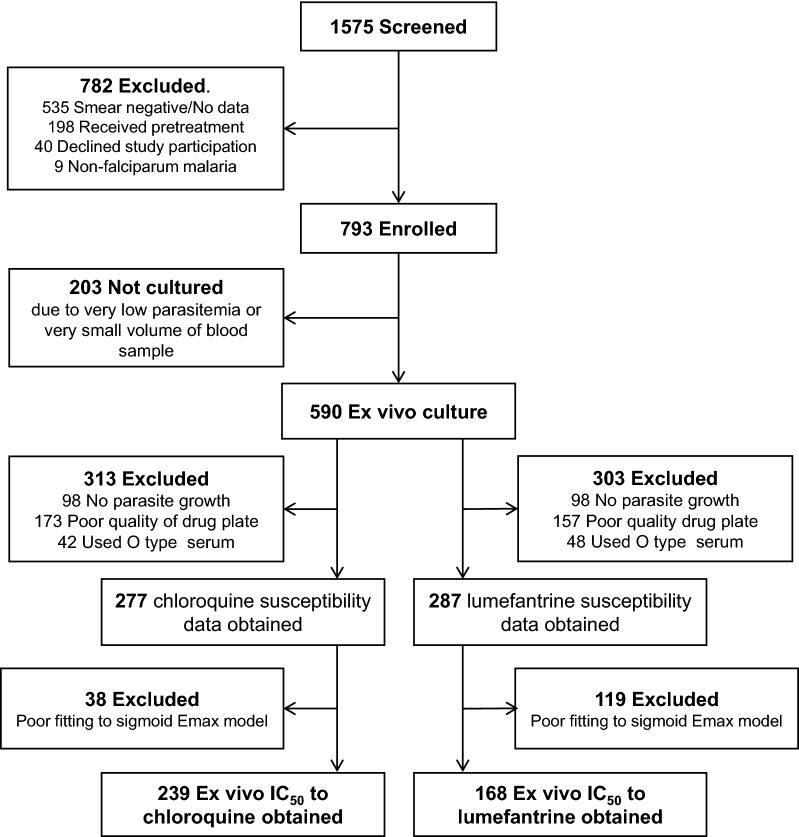
Flow chart of the study from screening to drug sensitivity assays, 2013–2018

Of 1575 patients who visited St. Mary’s Hospital Lacor, 793 patients were enrolled based on *P. falciparum*-positive results by RDT (Fig. 2). The rest were excluded because of (a) absence of *P. falciparum* by microscopic examination (n = 535), (b) use of anti-malarial drug(s) within the last 2 weeks before enrollment (n = 198), or (c) other reasons (n = 49) (Fig. 2). The commonly used anti-malarial for pretreatment was artemether–lumefantrine (77%) (Table 1). Chloroquine use was confirmed in only 3 patients in 2013 and one patient in 2014.

**Table 1:** Commonly used anti-malarial drugs of patients attending St. Mary’s Hospital Lacor, Gulu (information obtained from excluded patients)

Pretreatment drug, n (%)	2013 (N = 47)	2014 (N = 96)	2015 (N = 23)	2016 (N = 8)	2017 (N = 7)	Total (N = 181)
Artemether (intramuscular)	1 (2.1)	0	0	0	0	1 (0.6)
Artesunate (intravenous)	0	2 (2.1)	2 (8.7)	0	0	4 (2.2)
Chloroquine	3 (6.4)	1 (1.0)	0	0	0	4 (2.2)
Artemether–lumefantrine	34 (72.3)	67 (69.8)	6 (26.1)	7 (85.7)	6 (85.7)	120 (66.3)
Sulfadoxine–pyrimethamine	2 (4.3)	5 (5.2)	1 (4.4)	0	0	8 (4.4)
Quinine	6 (12.8)	6 (6.3)	1 (4.4)	1 (14.3)	0	14 (7.7)
Artesunate and artemether lumefantrine	0	1 (1.0)	0	0	0	1 (0.6)
Artemether–lumefantrine and sulfadoxine–pyrimethamine	0	1 (1.0)	0	0	0	1 (0.6)
Artemether–lumefantrine and quinine	0	3 (3.1)	0	0	0	3 (1.7)
Unknown	1 (2.1)	10 (10.4)	13 (57.0)	0	1 (14.3)	25 (13.8)

Among 793 blood samples obtained from enrolled patients, 203 were excluded because of very low parasitaemia (< 0.05%) or insufficient amount of blood, resulting in 590 samples used for ex vivo drug susceptibility assays. Also, ex vivo study for chloroquine in 2016 and lumefantrine in 2015 was not performed because of inadequate quality of pre-dosed drug plates. Thus, in total, 319 and 335 ex vivo drug-susceptibility assays for chloroquine and lumefantrine respectively were available for analyses. Background information of patients who participated in the study per year is shown in Table 2. Median age was 3.5 years (IQR 2.0–4.8) and hemoglobin level < 10 g/dL was observed in 27% of patients. Median parasitemia at enrollment were 0.2–3.5%, which significantly varied between studied years (p-value = 3.9 × 10^−15^, Kruskal–Wallis test). Beside parasitaemia at enrollment, no significant difference was observed in background factors among the studied years.

**Table 2:** Characteristics of participants evaluated for chloroquine and lumefantrine ex vivo susceptibility assay

Characteristic	2013 (N = 18)	2014 (N = 49)	2015 (N = 69)	2016 (N = 24)	2017 (N = 70)	2018 (N = 33)
Median age, years (IQR)	5.0 (2.3, 7.3)	3.4 (2.0, 5.8)	4.6 (2.4, 4.8)	3.1 (2.0, 5.5)	2.5 (1.1, 3.8)	3.5 (1.0, 4.7)
Gender (male/female)	8/10	28/21	30/38	14/10	33/37	17/16
Clinical findings (n, %)						
Muscle or joint aches	ND	3 (6.1)	3 (4.4)	2 (8.3)	1 (1.4)	3 (9.1)
Headache	ND	6 (12.2)	8 (11.6)	3 (12.5)	5 (7.1)	8 (24.2)
Anorexia	ND	11 (22.5)	7 (10.1)	1 (4.2)	5 (7.1)	12 (36.4)
Nausea/vomiting	ND	9 (18.4)	17 (24.6)	11 (45.8)	20 (28.6)	17 (51.5)
Abdominal pain	ND	6 (12.2)	7 (10.1)	0	3 (4.3)	0
Diarrhoea	ND	5 (10.2)	6 (8.7)	5 (21.0)	7 (10.0)	5 (15.2)
Cough	ND	18 (37.0)	10 (14.5)	6 (25.0)	21 (30.0)	28 (84.9)
Convulsions	ND	1 (2.0)	1 (1.5)	2 (8.3)	3 (4.3)	2 (6.1)
Jaundice	ND	2 (4.1)	0	0	0	0
Temperature, °C (IQR)	37.8 (37.4, 38.6)	38.1 (37.2, 39.0)	38.0 (37.5, 39.0)	37.7 (37.5, 39.3)	38.8 (37.8, 39.4)	38.5 (37.9, 38.9)
Laboratory findings						
Haemoglobin, g/dL (Range)	ND	10.6 (6.3–13.1)	ND	ND	12.7 (11.2–14.1)	10.5 (9.3–11.6)
Parasitaemia*, % (IQR)	0.2 (0.1, 0.5)	1.0 (0.2, 2.6)	2.1 (0.7, 4.8)	2.1 (1.3, 3.3)	3.5 (2.4, 6.0)	2.0 (1.5, 2.8)

*ND* not done, *IQR* inter quartile range

* Significant differences were observed between studied year (p-value = 3.9 × 10^−15^, Kruskal–Wallis test)

**Fig. 3:**
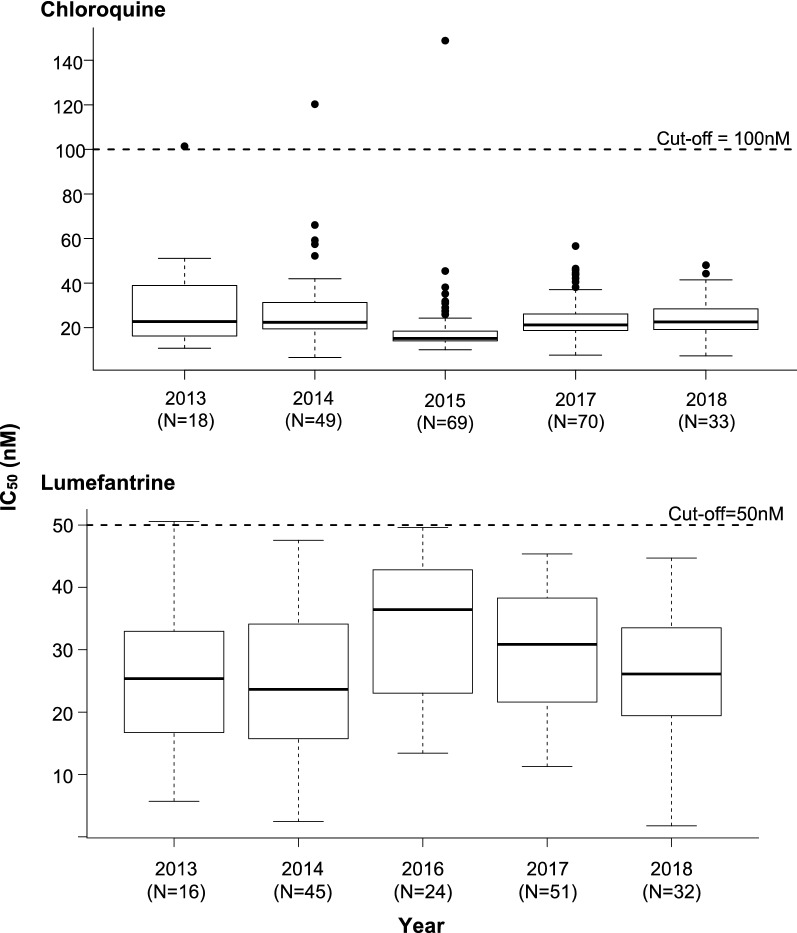
Ex vivo sensitivity of *P. falciparum* to chloroquine and lumefantrine. Bold lines represent median IC_50_s. Faint horizontal lines represent the 25th and 75th interquartile range. Mean IC_50_ for chloroquine were 24.8 nM, 24.9 nM, 17.4 nM, 22.6 nM and 23.1 nM in 2013, 2014, 2015, 2017 and 2018, respectively; and for lumefantrine, 20.8 nM, 20.5 nM, 32.0 nM, 28.6 nM and 21.0 nM in 2013, 2014, 2016, 2017 and 2018, respectively. Cut-off sensitivity is based from literature

Of 319 and 335 ex vivo drug-susceptibility assay for chloroquine and lumefantrine, respectively, 42 chloroquine and 48 lumefantrine assays were performed with O instead of AB blood-group serum due to the latter’s unavailability during the sampling period. These samples were excluded from further analysis. Thus, in summary, ex vivo drug study was successfully conducted for 239/277 samples (86.3%) for chloroquine and 168/287 for lumefantrine (58.5%) (Fig. 2). For chloroquine, only 1.3% (3/239) fulfilled the criteria for chloroquine resistance (IC_50_ > 100 nM) (Fig. 3a). From 2013 to 2018, the geometric means of the IC_50_s (17.4–24.9 nM) were much lower than the threshold for chloroquine resistance and was stable without any significant decrease or increase in trend throughout the study period (p-value = 0.32 Jonckheere-Terpstra test). The highest IC_50_ was 148.8 nM, observed in 2015 in a 9-year-old girl. For lumefantrine, IC_50_s displayed no specific trend over time ranging from 20.5 nM to 32.0 nM (p-value = 0.16, Jonckheere-Terpstra test). In all studied parasites, IC_50_ values were below the conservative cut-off of 50 nM for lumefantrine resistance [33], and lower than the 150 nM value [34] (Fig. 3b).

### Allele prevalence and frequency of *pfcrt* and *pfmdr1* polymorphisms

**Fig. 4:**
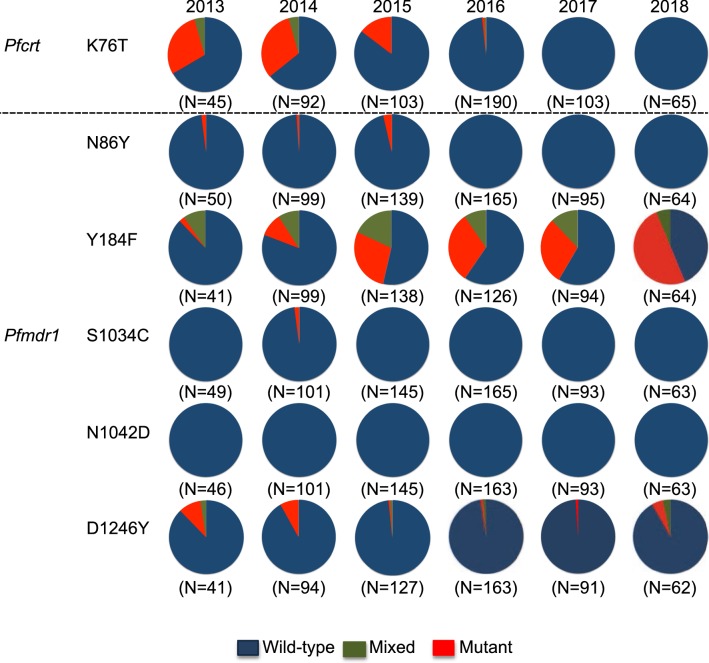
Temporal changes in *pfcrt* and *pfmdr1* allele prevalence in all collected *P. falciparum* isolates

In *pfcrt*, the prevalence of chloroquine-resistant alleles (CVIET allele; amino acid position 72–76, mutation underlined) significantly decreased from 28.8% in 2013 to 1.1% in 2016 and finally could not be detected in 2017 (Fig. 4). Besides genome sequencing, the absence of minor alleles bearing CVIET was further confirmed by *pfcrt* target sequencing. In *pfmdr1*, chloroquine sensitive N86 allele was fixed or nearly fixed throughout the study period. Prevalence of the mutant allele at position 184 (Y184F) gradually increased from 2.4% in 2013 to 48.5% in 2018, albeit this trend was not significant (p-value = 0.13, Jonckheere-Terpstra trend test). Wild-type alleles were nearly fixed at other loci in *pfmdr1*.

### Association between ex vivo drug sensitivity and alleles in *pfcrt* and *pfmdr1*

**Fig. 5:**
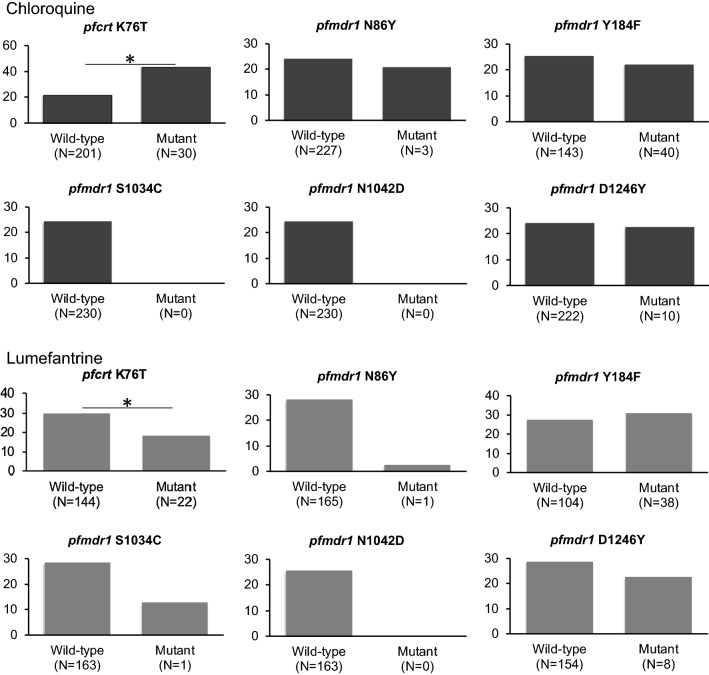
Association between allele prevalence and mean IC_50_ to chloroquine and lumefantrine. N is the number of samples available for comparison. Allele prevalence was compared between wild-type and mutant alleles using Wilcoxon rank sum test, and comparisons with *p* values of < 0.05 are significant. *p* values < 0.0001 are indicated by *

In *pfcrt*, parasites carrying wild-type alleles showed significantly lower IC_50_s to chloroquine than those carrying mutant allele (geometric mean, 21.4 vs. 43.1 nM, p-value 3.9 × 10^−8^, Wilcoxon rank sum test) (Fig. 5). To see whether other polymorphism(s) besides those at position 72–76 played a role in the recovery of chloroquine susceptibility, entire sequences of *pfcrt* in 56 samples were analysed. IC_50_s were also successfully obtained in 44 samples (Table 3), in which 31 (71%) bore only wild-type alleles of the gene. The second (n = 5, 11%) most common haplotype, HP-4, corresponds to a prevalent mutant haplotype (CVMNT + A220S + Q271E + R371I) in Africa [3, 35]. Remaining eight parasites harbored minor haplotypes, all with wild type allele K76 and displayed IC_50s_ to chloroquine of 18–35 nM. A previous study (16), has implicated C356R in the sensitivity recovery of chloroquine in the K76T-harbouring parasites, however this mutant allele was not found in the study area. These results further confirm the expansion of wild K76-harbouring parasites as the cause of chloroquine susceptibility reversal in the studied area, rather than additional mutation in the *pfcrt* gene.

**Table 3:** Chloroquine susceptibility in *pfcrt* haplotypes obtained from sequencing the entire *pfcrt* gene

*pfcrt* haplotypes	*pfcrt* polymorphic positions										Mean chloroquine IC_50_
	24	34	72	73	74	75	76	220	271	371	
HP 1 (n = 44)	D	R	C	V	M	N	K	A	Q	R	18.2 (n = 31)
HP 2 (n = 4)	Y	R	C	V	M	N	K	A	Q	R	22.2 (n = 4)
HP 3 (n = 1)	D	G	C	V	M	N	K	A	Q	R	19.7 (n = 1)
HP 4 (n = 5)	D	R	C	V	I	E	T	S	E	I	22.2 (n = 5)
HP 5 (n = 2)	D	R	C	V	M	N	K	A	E	R	35.2 (n = 2)
HP 6 (n = 1)	D	R	C	V	M	N	K	A	Q	I	17.5 (n = 1)

Association between ex vivo drug sensitivity and respective *pfmdr1* alleles, however, could not be properly evaluated because of substantial deviation in allele frequencies except for position 184. At this position, no significant difference in chloroquine IC_50s_ was observed between wild and mutant alleles (25.3 nM vs. 22.0 nM, p-value = 0.192, Wilcoxon rank sum test) (Fig. 5). Analysis of susceptibility to lumefantrine indicated that parasites carrying *pfcrt* CVIET exhibited significantly lower IC_50_s than those with CVMNK (13.4 vs. 28.0 nM, p-value = 8.1 × 10^−5^, Wilcoxon rank sum test) (Fig. 5). No significant difference in IC_50_s for lumefantrine was found between parasites with *pfmdr1* Y184 (25.0 nM) and Y184F (30.0 nM).

## Discussion

In Uganda, chloroquine was officially withdrawn and replaced with artemether–lumefantrine in 2006. The present analysis revealed that chloroquine susceptibility has returned and is stably maintained for at least 6-years in Gulu, Northern Uganda. This is the first report in Uganda to demonstrate stable and persistent recovery of chloroquine sensitivity using both phenotypic and genotypic approaches.

The prevalence of K76 allele in *pfcrt* rapidly increased from 67% in 2013 to complete fixation in 2017. This is most probably due to K76-harbouring parasites outcompeting the K76T-harbouring parasites because of fitness advantage in the absence of chloroquine selection pressure, as previously observed in Malawi [36, 37]. Indeed, recent transfection studies also show that K76T confers a substantial fitness cost to parasites [35, 38]. This fitness cost can be partly explained by a functional impairment in haemoglobin digestion and subsequent reduction in the supply of amino acids in K76T harbouring parasites [39]. In another possible scenario, a back mutation from T to K at position 76 in *pfcrt* may potentially induce chloroquine sensitivity. As an example, the chloroquine-susceptible 106/1 clone had mutant alleles at positions 74 and 75 but showed wild-type K allele at position 76 (CVIEK) [40]. The only difference in the *pfcrt* haplotype at position 72–76 in this clone and the widely prevalent chloroquine-resistant haplotype (CVIET) is at amino acid position 76. In the present analysis, however, no such haplotype (CVIEK) was found, negating this possibility. Also, no evidence of additional mutation in *pfcrt* such as C350R which has been reported to be associated with the restoration of chloroquine susceptibility [16] was obtained. Taken together, these results strongly suggest that neither back mutation nor additional mutations in *pfcrt* was associated with the observed recovery of chloroquine sensitivity in the study area.

It is of note that recovery of chloroquine sensitivity after its withdrawal occurred much earlier in Gulu than in other regions in Uganda [9, 22–24, 41]. In 2013, as much as 65% of parasites displayed ex vivo chloroquine resistance [24] and 60–80% carried K76T allele in Tororo, Eastern Uganda [42]. In contrast, present results revealed that prevalence of ex vivo chloroquine-resistance and K76T alleles were already 6% and 29%, respectively, in 2013, indicating a faster recovery or re-emergence of chloroquine sensitive strains in the region. Despite the government’s move to change the national treatment policy, chloroquine might be used as self-treatment and/or prophylaxis. Such chloroquine use potentially creates various levels of chloroquine selecting pressure in the region, which would be one of the important factors that affects the speed of recovery of susceptible parasites [43]. However, countrywide anti-malarial survey reported no considerable difference of chloroquine use between Tororo and this study area [44–46], suggesting that this could not be a main factor for the observed findings.

Piperaquine use in Tororo might explain the observed difference to some extent. Dihydroartemisinin-piperaquine has been used as a second line treatment for uncomplicated malaria in Uganda. In Tororo, this regimen was widely used in different drug trials for malaria treatment [47, 48] and chemoprevention [24, 49–52]. Previous studies indicated that dihydroartemisinin-piperaquine treatment selected the N86Y allele in *pfmdr1* in Uganda [48, 52–54], albeit one study questioned this association [55]. Approximately one-third of parasites possessed the N86Y in 2010–2013 in Tororo [9], which was much higher than that found in the studied area (2%). Since N86Y is associated with chloroquine resistance, it might be plausible that the N86Y mutation selected by piperaquine played a role in the slower recovery of chloroquine sensitivity in Tororo.

In contrast to chloroquine, average lumefantrine IC_50_ values in the present analysis (21–29 nM) were considerably higher than those in Eastern Uganda (3.0–5.4 nM) [9, 24]. It has been suggested that decrease in lumefantrine susceptibility are associated with wild type alleles in *pfcrt* and *pfmdr1* [48, 56–59]. In this study higher IC_50_s to lumefantrine were observed in K76 sequences than those with K76T. The higher prevalence of K76 allele in our study area than that in Eastern Uganda [9, 23, 42] may partly explain the observed lumefantrine susceptibility.

Molecular epidemiological analysis displayed a large increase in the proportion of parasites carrying the Y184F mutation in *pfmdr1*. This occurred at the same time when substantial reduction on the *pfcrt* K76T mutation was observed, particularly between 2014 and 2015. One possible explanation would be an increase in lumefantrine usage results in the selection of these alleles in this area. Indeed, previous in vivo studies have demonstrated that artemether–lumefantrine treatment selected for these alleles (*pfcrt* K76 and Y184F) in Africa including Uganda [24, 60, 61]. In the present study, IC_50_s for lumefantrine were significantly higher in *pfcrt* K76 harboring parasites than those harboring K76T; while, Y184F allele did not show significantly high IC_50_s for lumefantrine, consistent with previous transfection study that revealed no association of Y184F mutation to in vitro lumefantrine susceptibility [59]. Thus, in vivo selection for Y184F allele after artemether–lumefantrine treatment may be because of mechanisms other than susceptibility to lumefantrine.

## Conclusions

The study shows the stable persistence of chloroquine-susceptibility with the fixation of *pfcrt* K76 in Northern Uganda. This observation implies the possibility of future clinical trials for potential re-use of chloroquine as an option for malaria treatment or prevention. Such trial was performed in Malawi where longtime stable chloroquine susceptibility has been evidenced and has revealed that weekly chemoprophylaxis with chloroquine showed 78% lower risk of clinical malaria than intermittent sulfadoxine-pyrimethamine [62]. Similar trial in Uganda would provide an insight into potential re-introduction of chloroquine. However, further evidence of long-time persistence of return of chloroquine susceptibility is warranted in other endemic areas in Uganda before the implementation of clinical trials.

## Data Availability

The primary datasets used and analyzed during the current study are available from the corresponding author on reasonable request.

## Abbreviations

ACT: Artemisinin-based combination therapy
DDBJ: DNA data bank of Japan
DFID: Department for international development (UK)
DNA: Deoxyribonucleic acid
ELISA: Enzyme-linked immunosorbent assay
HRP-2: Histidine-rich protein-2
IC_50_: 50% growth inhibitory concentration
IRS: Indoor residual spray
LLIN: Long-lasting insecticidal mosquito net
*pfcrt*: *Plasmodium falciparum chloroquine resistance transporter gene*
*pfmdr1*: *Plasmodium falciparum multidrug resistance*-*1*
PCR: Polymerase chain reaction
PMI: President’s malaria initiative
RDT: Rapid diagnostic test
USAID: United States Agency for International Development
